# Network meta-analysis of heat-clearing and detoxifying oral liquid of Chinese medicines in treatment of children's hand-foot-mouth disease: A protocol for systematic review and meta-analysis

**DOI:** 10.1097/MD.0000000000028778

**Published:** 2022-02-04

**Authors:** Jiqin Tang, Gong Zhang, Jinxiao Xing, Ying Yu, Tao Han

**Affiliations:** a College of Continuing Education, Shandong University of Traditional Chinese Medicine, Jinan, China; b College of Integrated Traditional Chinese and Western Medicine, Shandong Liming Vocational College of Science and Technology, Tai’an, China; c College of Rehabilitation Medicine, Shandong University of Traditional Chinese Medicine, Jinan, China; d Innovative Institute of Chinese Medicine and Pharmacy, Shandong University of Traditional Chinese Medicine, Jinan, China; e Graduate Office of Shandong University of Traditional Chinese Medicine, Jinan, China.

**Keywords:** Chinese patent medicine oral liquid, hand-foot-mouth disease, network meta-analysis, protocol, ribavirin

## Abstract

**Background:**

Hand-foot-mouth is a viral infectious disease characterized by fever, hand foot rash and oral mucosal herpes caused by a variety of enteroviruses. It is often found in preschool children, and its immune system is not well developed, so it is very susceptible to infection by pathogens and epidemics, resulting in rapid progress of the disease. At present, the commonly used Chinese patent medicine oral liquid in our country has good clinical efficacy of antiviral, antibacterial, antiphlogistic and improving immunity, but there is no evidence to compare the clinical efficacy and safety of a variety of oral liquid of Chinese patent medicine. Therefore, this study is aim to use the network meta-analysis to integrate the clinical relevant evidence of direct and indirect comparative relationship, and to conduct quantitative comprehensive statistical analysis and sequencing after the aggregation of different Chinese patent medicine oral liquid with the same evidence body, and then the best clinical medication scheme is selected, which can provide reference value and evidence-based theoretical evidence for clinical optimization of drug selection.

**Methods:**

Comprehensive retrieval of CNKI, VIP, CBM, and WANFANG database and the Cochrane Library, PubMed, Web of Science and EMBASE database. Search and publish the clinical RCT of these 7 kinds of oral liquid of Chinese patent medicine compared with ribavirin or oral liquid of Chinese patent medicine. The retrieval time is from the establishment of the database to October 31st, 2021. The 2 first authors will screen the literatures that meets the inclusion criteria, extract the data independently according to the predesigned rules, and evaluate the literature quality and bias risk of the included research according to the Cochrane manual standard. Data merging and network meta-analysis were carried out with R programming software to evaluate the ranking probability of all interventions.

**Results:**

This network meta-analysis and probability ranking will identify the best Chinese patent medicine oral liquid treatment for Hand-foot-mouth.

**Conclusion:**

This study will provide systematic evidence-based medicine evidence for Chinese patent medicine oral liquid treatment for Hand-foot-mouth, and help clinicians, patients with poststroke depression and decision-makers to make more effective, safer and economic optimal treatment plan in the decision-making process.

**Registration number:**

INPLASY202210032. The protocol for this systematic review was registered on INPLASY and is available in full on the inplasy.com (https://inplasy.com/inplasy-2022–1-0032/).

## 1 Introduction

Hand-foot-mouth disease (HFMD) is an infectious disease caused by a variety of enteroviruses (entero Coxsackie virus, enterovirus-71-Cox A16 and EV71), which is a national legal class C infectious disease.^[1]^ Patients and invisible patients are the source of infection of HFMD. Close contact is an important mode of transmission of HFMD, which can be transmitted through the respiratory tract through droplets. The disease is mostly found in preschool children under 5 years old. The disease is highly infectious, spreads rapidly, and causes outbreaks in densely populated areas. Most of the clinical symptoms were fever, herpes or ulcer in oral mucosa, maculopapular rash in palms, soles and other skin parts, and then turned to herpes. The pathogenesis of the disease often presents the characteristics of diversity, outbreak, infectivity, epidemic and seasonality, and a few patients with pain will cause myocarditis, meningitis, encephalomyelitis, neuritic pulmonary edema and other relatively fatal complications. At the same time, some severe children will also have respiratory distress syndrome, cardiac decline, central nervous system disorder, so it is necessary to prevent and intervene as early as possible.^[2]^ Western medicine mostly adopts symptomatic treatment, enterovirus control, bacterial infection prevention and other methods in the treatment of the disease. Most of them choose broad-spectrum anti-virus and anti infective drugs, and there is no specific drugs such as anti enterovirus. At present, ribavirin, a synthetic nucleoside antiviral drug, is widely used in clinic. Its mechanism of action is that it can directly affect virus infected cells, inhibit DNA polymerase activity, and inhibit the synthesis of protein by RNA. Although it can effectively alleviate clinical symptoms, it is easy to produce drug resistance and adverse reactions, which will seriously affect the efficacy of clinical diagnosis and treatment and the safety of children's treatment, so that its clinical application is greatly limited.

Traditional Chinese medicine, as an important part of complementary and alternative medicine, has played a huge advantage in the research of antiviral treatment. Many clinical practices have confirmed that a variety of oral liquid of Chinese patent medicine developed from natural herbs has been widely used in the treatment of children's viral hand-foot-mouth disease, which can not only give full play to the clinical efficacy of antiviral, but also effectively improve the body immunity of children. At the same time, it can select oral liquid preparation for young children to take, which is not only stable, simple, safe and reliable, low price, but also convenient and oral sensory optimization and other characteristics can greatly improve the subjective initiative and treatment compliance of clinical patients, so it has a certain clinical application value. Therefore, the author selected 7 kinds of oral liquid of Chinese patent medicine, which are most commonly used in clinic, to treat children's viral hand, foot and mouth disease. They are respectively Pudilan oral liquid, Lanqin oral liquid, Huangqin oral liquid, Shuanghuanglian oral liquid, huangzhihua oral liquid, antiviral oral liquid and Fuganlin oral liquid. The control group selected western medicine ribavirin or one of the 7 kinds of oral liquid of Chinese patent medicine as the contrast treatment. At present, there are 2 comparative clinical efficacy studies on the treatment of HFMD by oral liquid of Chinese patent medicine at home and abroad. There is no comparison on the clinical efficacy and safety of multiple oral liquid of Chinese patent medicine in the treatment of HFMD in children. Therefore, the purpose of this study is to use the network meta-analysis method to integrate the clinical relevant evidence of direct and indirect comparative relationship, to make quantitative comprehensive statistical analysis and sequencing of different oral liquid of traditional Chinese medicine with the same evidence body for the treatment of the disease, and then to explore the advantages and disadvantages of the efficacy and safety of different oral liquid of traditional Chinese medicine to get the best treatment plan, so as to provide reference value and evidence-based medicine evidence for clinical optimization of drug selection.^[3–5]^

## 2 Methods

### 2.1 Protocol and registration

We will complete this protocol for systematic evaluation and network meta-analysis, which follows the statements of the Cochrane manual standard, the Grading of Recommendations Assessment, Development and Evaluation(GRADE) and “protocol for systematic evaluation and network meta-analysis” in accordance with recognized standards. A report on the further results of this study will be submitted in accordance with the guidelines of the PRISMA network meta-analysis extension statement. We have obtained the registration number (INPLASY202210032) of this study on the platform of INPLASY (https://inplasy.com/inplasy-2022-1-0032/). Because the network meta-analysis protocol has been approved by the local agency review committee and the ethics committee, it does not involve privacy information and does not require further ethical approval and informed consent.

### 2.2 Information sources

By using the computer retrieval technology, the literature retrieval of the clinical randomized controlled study on the treatment of children's viral hand, foot and mouth disease with 7 kinds of Chinese patent medicine oral liquid was carried out. The primary search was selected and the set period was from the establishment of the database to October 31st, 2021. The computer retrieval electronic database included CNKI, CBM, WANFANG data, VIP, and other Chinese databases, as well as the Cochrane Library, PubMed, Web of Science and EMBASE and other foreign databases. Chinese search words include: hand-foot-mouth disease, Chinese patent medicine, Pudilan oral liquid, Lanqin oral liquid, Huangqin oral liquid, Shuanghuanglian oral liquid, huangzhihua oral liquid, antiviral oral liquid, Fuganlin oral liquid, ribavirin, random, etc. English search words include: (Hand-foot-mouth OR Hand, Foot and Mouth Disease OR Hand, Foot, Mouth Disease OR HFMD) and (Pudilan oral liquid OR Lanqin oral liquid OR Huangqin oral liquid OR Shuanghuanglian oral liquid OR Huangzhihua oral liquid OR Antiviral oral liquid OR Fuganlin oral liquid) and (Ribavirin) and (Random∗OR randomized controlled trials OR clinical randomized controlled trials).

When searching the literature, the subject words and free words shall be searched separately, and the relevant free words and terms shall be used for comprehensive search. Meanwhile, the research of WHO international clinical trial registration platform and ClinicalTrials.gov shall be searched to determine the additional potential trial registration. In addition, the relevant journals shall be searched in the reference literature, and the relevant literature shall be tracked, and Google scholars shall be used together Baidu academic and other relevant search engines conduct relevant research on the Internet by hand, and will provide data for all relevant authors and major researchers to supplement the incomplete report or unpublished research of the original paper. We will try our best to ensure that the primary search work is comprehensive so as not to lose valuable research materials. At the same time, according to the Participant-Intervention-Comparator-Outcomes-Study design search principle, we will include the research that meets the standards and organize and create the database. In Table 1, the preliminary search strategy of PubMed database is taken as an example to summarize the preliminary search strategy, which will be adjusted according to the requirements of other electronic databases related to keywords.

**Table 1:** Search strategy in PubMed database.

Number	Search terms
#1	Search “Hand-foot-mouth Disease” [Mesh]
#2	Search (((Hand, Foot, Mouth Disease [Title/Abstract]) OR (Hand, Foot [Title/Abstract] AND Mouth Disease [Title/Abstract])) OR Hand, Foot, Mouth Disease [Title/Abstract]) OR HFMD [Title/Abstract]
#3	#1 OR #2
#4	Search “Oral liquid”[Mesh]
#5	Search ((Oral liquid[Title/Abstract]) OR Oral solution [Title/Abstract])
#6	Search (((((((Pudilan oral liquid [Title/Abstract]) OR Lanqin oral liquid [Title/Abstract]) OR Huangqin oral liquid [Title/Abstract]) OR Shuanghuanglian oral liquid [Title/Abstract]) OR Huangzhihua oral liquid [Title/Abstract]) OR Antiviral oral liquid [Title/Abstract]) OR Fuganlin oral liquid [Title/Abstract]
#7	#4 OR #5 OR #6
#8	Search “Ribavirin” [Mesh]
#9	Search (((((((((((((Ribovirin[Title/Abstract]) OR Tribavirin [Title/Abstract]) OR Rebetol [Title/Abstract]) OR Virazole [Title/Abstract]) OR Vilona [Title/Abstract]) OR Ribasphere [Title/Abstract]) OR Viramide [Title/Abstract]) OR Virazide [Title/Abstract]) OR ICN-1229 [Title/Abstract]) OR ICN 1229 [Title/Abstract]) OR ICN1229 [Title/Abstract]) OR Ribamide [Title/Abstract]) OR Ribamidil [Title/Abstract]) OR Ribamidyl [Title/Abstract]
#10	#8 OR #9
#11	Search “Randomized Controlled Trial” [Publication Type]
#12	Search (((((Controlled Clinical Trial [Title/Abstract]) OR Randomized [Title/Abstract]) OR Placebo [Title/Abstract]) OR Randomly [Title/Abstract]) OR Trial [Title/Abstract]) OR Groups [Title/Abstract]
#13	#11 OR #12
#14	#3 AND #7 AND #10 AND #13

This search strategy will be modified as required for other electronic databases.

### 2.3 Eligibility criteria

The design of inclusion criteria and exclusion criteria in this study is based on the 5 main principles of Participant-Intervention-Comparator-Outcomes-Study design.

#### 2.3.1 Type of participant

The patient is a single viral HFMD child patient, whose gender, race and region are not limited. The diagnostic criteria of HFMD used shall conform to 1 of the following: guidelines for diagnosis and treatment of HFMD (2010 Edition) or Zhu Futang Practical Pediatrics (Seventh Edition) and diagnostic efficacy criteria of TCM disease syndromes, and the diagnosis of TCM syndrome type shall conform to the criteria of HFMD in the seventh edition of Practical Pediatrics or the second edition of Pediatrics of TCM.^[6,7]^

#### 2.3.2 Type of interventions and comparators

When there are clear diagnostic criteria, curative effect judgment criteria and basic treatment are consistent, one of the Chinese patent medicine oral liquid (Pudilan oral liquid or Lanqin oral liquid or Huangqin oral liquid or Shuanghuanglian oral liquid or Huangzhihua oral liquid or Anti-virus oral liquid or Fuganlin oral liquid) is used as the intervention measure of the experimental group. In the control group, ribavirin alone or one of the 7 kinds of oral liquid of Chinese patent medicine was selected as the intervention measures, and no other combination drugs were used.

#### 2.3.3 Type of outcomes

The evaluation indexes are mainly divided into the main outcome indexes and the secondary outcome indexes, among which the main outcome indexes include: Clinical total effective rate, Cure rate and Adverse reactions. The secondary outcome indexes include: Healing time of oral ulcer, Disappearance time of hand and foot rash, Hospitalization time, Antipyretic time and other related disease symptom score comparison information acquisition.

#### 2.3.4 Type of study

The literature included were Randomized Controlled Trials with no limitation on language and blind or assignment concealment. As long as the Chinese trial is approved by the local institutional review committee and registered in the international database, we will include its research into the scope of the study. In addition, the author will delete nonrandomized controlled trials, case reports, experience summaries, self-control, and comprehensive literature; animal experimental studies; simple descriptive literature, repeatedly published literature, literature with unclear diagnosis of hand, foot and mouth disease in children or other diseases, the efficacy judgment criteria of the trial group and the control group are not clear, the baseline treatment measures are inconsistent, and the treatment measures are not clear Literature related to the causal relationship interpretation of other treatment methods affecting the final treatment, literature with unclear research results and incomplete data.

### 2.4 Selection of studies

**Figure 1:**
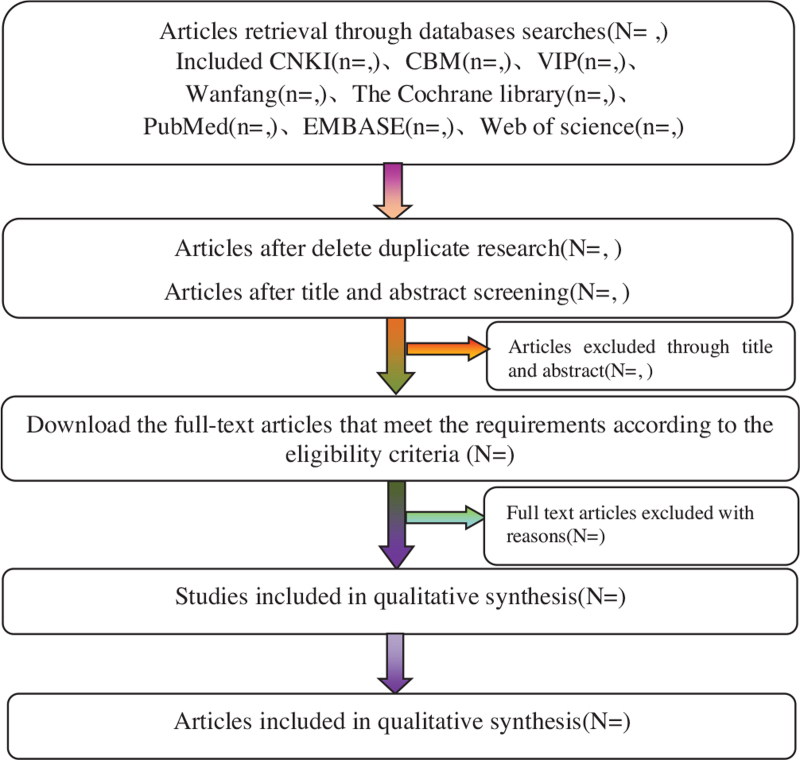
Flow diagram of study.

According to the retrieval strategy of the above electronic databases, 2 researchers searched the electronic databases in Chinese and English, used Endnote X9 software to search the repeated information, combined the literature retrieval results in different databases, established the information database and downloaded the full text. Then 2 first authors independently extract the data for preliminary screening, extract the data according to the predetermined table, take cross check and review, and mark down the reasons for each excluded study, invite the third review researcher to jointly discuss and make a final decision on the research with different opinions. The process of study selection will be summarized in the PRISMA flowchart in Figure 1.^[8,9]^

### 2.5 Data extraction and management

Data extraction content includes

1. Basic information of the included literature (including the first author, published journal and year, research topic).
2. Relevant information of the treatment group and the control group in the literature (including the number of cases, total cases, age, intervention measures, course of treatment, outcome indicators).
3. Design type and quality evaluation information of the included literature.
4. Outcomes (The main outcome indexes include: Clinical total effective rate, Cure rate and Adverse reactions. The secondary outcome indexes include: Healing time of oral ulcer, Disappearance time of hand and foot rash, Hospitalization time, Antipyretic time and other related disease symptom score comparison information acquisition).

### 2.6 Study quality evaluation

According to the quality evaluation standard of Cochrane system evaluator manual, Revman quality evaluation tool was used to evaluate the methodological quality of the included study, including random method, assignment concealment, blind method, outcome data integrity, selective report, number of dropped cases, follow-up and other biases. Each project was divided into 3 options: high risk, low risk and uncertainty risk, According to the description of the above aspects in the included research, the 2 first authors independently completed the quality evaluation results of the included literature. If there are differences in the results, it is necessary to invite the third researcher to help each other discuss and make interpretation and quality evaluation. According to the standards of Cochrane manual, literature quality assessment and bias risk assessment were carried out. Statistical software, data integration and network meta-analysis are carried out by R language programming software.^[10]^

Consulting GRADE handbook, the assessment which would be carried out through the Grading of Recommendations Assessment, Development, and Evaluation (GRADE, https://gradepro.org/) by 2 independent authors will be designated into 4 grades: high quality, moderate quality, low quality, and very low quality.^[11]^

### 2.7 Data synthesis and statistical methods

#### 2.7.1 Pairwise and network meta-analysis

Revman software is provided by Cochrane collaborative network for literature quality and bias risk assessment. R language programming software is used for direct and indirect results comparison and 95% confidence interval (CI) calculation in network meta-analysis. At the same time, network relationship diagram and anecdotal sequence diagram of various interventions are drawn, which can effectively show the indirect comparison relationship of 7 kinds of proprietary Chinese medicine oral liquids. The network diagram is mainly composed of nodes and Line composition, in which node represents a treatment mode, the node connected by a straight line represents a direct or indirect comparative relationship between the 2, and the thickness of the connecting line represents the number of studies. Then, we will analyze the results of all direct or indirect comparisons to evaluate which is the best treatment plan for children with viral HFMD treated by these 7 kinds of Chinese patent medicine oral liquid, and estimate the rank probability of each group based on Markov chain Monte Carlo method. R programming language starts “NETMETA” program, and calls Bayes Markov China Monte Carlo algorithm to analyze the data of random effect model. The OR value (Odd Ratio) was used as the statistical value of efficacy analysis, the weighted mean difference or standardized mean difference was used as the measurement data, and 95% CI was used for each effect. Odd Ratio was used as the statistical measure of effective rate, healing time of related symptoms and adverse reactions, and 95% CI confidence intervals was used to express the effect. Based on the network meta probability ranking, in the main outcome indicators, the larger the probability *P* value of clinical total effective rate and cure rate, the better the probability *P* value of adverse reaction rate. In the information of symptom score comparison of related diseases in secondary outcome indicators, the smaller the *P* value is, the better the healing time of oral ulcer, the time of resolving rash of hands and feet, the time of antipyretic, the time of hospitalization, etc.^[12,13]^

#### 2.7.2 Assessment of heterogeneity

Heterogeneity will be evaluated by Cochrane. For each pairing comparison, statistical heterogeneity will be evaluated by *I*^***2***^ index, subgroup analysis based on the heterogeneity factors and study by **χ**^**2**^ test. Evaluate the clinical and method heterogeneity of the included study, and compare the fitting degree of fixed effect model and random effect model. If each study in the subgroup has statistical homogeneity (*P* ≥ .1, *I*^***2***^ ≤ 50%), the fixed effect mode is used for meta-analysis. Otherwise, the causes of heterogeneity are analyzed first, and the random effect mode is used for meta-analysis without obvious clinical heterogeneity (*P* < .1, *I *^***2***^>* *50%), and the possible causes of heterogeneity are found out from both clinical and methodological aspects. If the clinical trial data provided cannot be meta-analyzed, descriptive analysis shall be conducted.

#### 2.7.3 Subgroup and sensitivity analyses

If the result of meta-analysis is positive and there are more than 3 included studies, R software shall be used to conduct sensitivity analysis on the statistical results, and meta-analysis shall be carried out again for each excluded study, and the results shall be compared with those before exclusion. If there is no substantial change in the comparative analysis, the results are stable. Otherwise, the data results are not stable. If significant heterogeneity is found, subgroup analysis will be envisaged based on treatment time, age, race, gender and quality of study to investigate possible sources of heterogeneity.^[14]^

#### 2.7.4 Assessment of inconsistency

The inconsistency between direct and indirect evidence will assess by using the node-splitting model, which can calculate the difference between direct and indirect evidence.^[15]^ We will determine if there is an inconsistency based on the *P* value. If there is no statistical difference (*P *>* *.05) in each study within the subgroup, it indicates that the heterogeneity of the included study is small, so the consistency model is used for analysis; otherwise, the inconsistent model is used for analysis.

#### 2.7.5 Publication bias

If more than 5 studies are included, R software is used to analyze the potential publication bias, and the figure is inverted funnel-shaped and symmetrical, which indicates that the possibility of publication bias is relatively small. If the figures are biased, it indicates that there is a greater possibility of publication bias.

## 3 Discussion

Traditional Chinese medicine treatment of this disease is generally based on clearing away heat, removing dampness and detoxification. The 7 kinds of oral liquid preparations of traditional Chinese medicine (Pudilan oral liquid, Lanqin oral liquid, Huangqin oral liquid, Shuanghuanglian oral liquid, huangzhihua oral liquid, antiviral oral liquid and fuganlin oral liquid) which are commonly used in clinical selected in this study are all made from the combination of pure natural Chinese herbal medicines of clearing away heat and detoxifying. This kind of preparation can not only effectively promote the compatibility of herbal medicine to play the role of anti-inflammatory, anti-bacterial, analgesic and antipyretic, but also improve the level of IgE and IgM of the body, regulate the immune function, promote the synthesis of the body's autoantibody to cure this disease, and also greatly shorten the time for the recovery of clinical related diseases. In terms of dosage form, Chinese patent medicine oral liquid preparation can not only avoid many inconveniences caused by herbal decoction, but also make use of sucrose auxiliary materials to adjust the taste so that it is very easy to be accepted by children in clinical. In addition, its clinical convenience, standardization, safety and other aspects continue to improve, which greatly promotes the clinical cure rate, shortening the course of disease, improving the safety and compliance of clinical children's treatment. Therefore, it has a certain clinical application value.

Under the condition that the syndrome types and oral dosage forms of traditional Chinese medicine are relatively consistent, the direct intervention treatment of 7 kinds of heat clearing and detoxifying oral liquid of traditional Chinese medicine have certain clinical comparability. However, there is no standardized diagnostic standard and therapeutic effect judgment standard for antiviral treatment of children's viral Hand-foot-mouth disease by traditional Chinese medicine. At present, most studies at home and abroad only focus on the clinical efficacy of the 2 comparative studies of traditional Chinese medicine oral liquid in the treatment of hand, foot and mouth disease, and there is still a lack of network meta-analysis on the clinical efficacy and safety to compare multiple traditional Chinese medicine oral liquid in the treatment of viral HAMD in the same clinical trial. Therefore, this study uses the network meta-analysis method to obtain the clinical total effective rate, cure rate, adverse reaction rate and the score comparison of the improvement of related symptoms and other outcome indicators, to clarify the antiviral efficacy of 7 kinds of traditional Chinese medicine oral liquid preparations, and then according to the advantages and disadvantages of the main indicators and secondary indicators of the efficacy of probability ranking, so as to effectively screen out the best clinical treatment options. The quality of these evidences will be evaluated again by the hierarchical method. Through a comprehensive analysis of the data, it can be said that it has a certain reference value for clinicians to select the oral liquid of traditional Chinese medicine.

However, there are certain defects in our Network Meta-Analysis, such as publication bias, clinical heterogeneity and selection bias, which will ultimately affect the recognition of the research results. While we still hope that this study can provide the best possible drug selection and reliable evidence-based medicine for clinical practice, and to some extent provide strong evidence for the significant advantages of traditional Chinese medicine in antiviral, so it can provide more reliable reference value for clinical practice.

**Ethics and dissemination:** This study will provide systematic evidence-based medicine evidence for the direct intervention of oral liquid of traditional Chinese medicine in the treatment of children's viral hand, foot and mouth disease, and help clinicians to make more effective, safer and economic optimal treatment plan in the decision-making process. This network meta-analysis protocol has been approved by the local institutional review committee and the ethics committee, further ethical approval and informed consent are not required, and the research results will be disseminated through peer-reviewed journal publications or academic conference reports. At present, the protocol for the network meta-analysis has been registered on the international system review expectation register (INPLASY202210032), which will follow the guidelines of “Cochrane Intervention System Review Manual” and “Prisma-P statement.” In addition, if the protocol needs to be amended, there will be a description of the amendment with the reason and the date.

## Author contributions

**Conceptualization:** Jiqin tang, Gong Zhang, Jinxiao Xing, Ying Yu.

**Data curation:** Jiqin Tang, Gong Zhang, Jinxiao Xing, Ying Yu.

**Formal analysis:** Jiqin Tang, Gong Zhang, Jinxiao Xing, Ying Yu.

**Funding acquisition:** Tao Han.

**Investigation:** Jiqin Tang, Gong Zhang, Ying Yu, Tao Han.

**Methodology:** Jiqin Tang, Gong Zhang, Jinxiao Xing, Ying Yu.

**Project administration:** Jiqin Tang, Gong Zhang, Jinxiao Xing, Ying Yu, Tao Han.

**Resources:** Jiqin Tang, Gong Zhang, Jinxiao Xing, Ying Yu.

**Software:** Jiqin Tang, Gong Zhang, Jinxiao Xing, Ying Yu.

**Supervision:** Ying Yu, Tao Han.

**Validation:** Ying Yu, Tao Han.

**Visualization:** Gong Zhang, Ying Yu, Tao Han.

**Writing – original draft:** Jiqin Tang, Gong Zhang, Ying Yu.

**Writing – review & editing:** Jiqin Tang, Gong Zhang, Ying Yu.
